# PBPK/PD Model of Vancomycin in Sepsis: Linking Interstitial Exposure in Perfusion-Limited Tissues to MRSA Infection

**DOI:** 10.3390/pharmaceutics17091111

**Published:** 2025-08-26

**Authors:** Laura Ben Olivo, Jéssica Luísa Silva de Lemos, Vinicius Jardim Rodrigues, Dúnia Batirola Kretschmer, William de Avila Cruz, Keli Jaqueline Staudt, Pieter Annaert, Bibiana Verlindo de Araújo

**Affiliations:** 1 Pharmacokinetics and PK/PD Modeling Laboratory, Pharmaceutical Sciences Graduate Program, Federal University of Rio Grande do Sul, 2752 Ipiranga Ave., Santana, Porto Alegre 90610-000, RS, Brazil; laura.olivo@ufrgs.br (L.B.O.); jllemos_@hotmail.com (J.L.S.d.L.); viniciusjrodrigues00@gmail.com (V.J.R.); 2 Department of Health Sciences, Integrated Regional University of Alto Uruguai and Missões, 464, St. Unisersidade das Missões, Santo Ângelo 98802-470, RS, Brazil; duuniabk@gmail.com (D.B.K.); willc6912@gmail.com (W.d.A.C.); kelijaquelines@gmail.com (K.J.S.); 3 Department of Pharmaceutical and Pharmacological Sciences, KU Leuven, 3000 Leuven, Belgium; pieter.annaert@kuleuven.be

**Keywords:** physiologically based modeling, PBPKPD, tissue interstitial concentration, MRSA infection

## Abstract

**Objective**: This study aims to evaluate free vancomycin concentrations in tissues of septic patients that received empirical doses. **Methods**: A PBPK model was built in PK-Sim to simulate vancomycin concentrations in healthy volunteers and septic patients. Literature data were used to validate the model. A strain of MRSA (methicillin-resistant *Staphylococcus aureus*) was evaluated through time-kill curves. Based on the information obtained from the time-kill study, a PD model, including adaptive resistance, was developed using NONMEM. The PBPK and PD models were combined to evaluate the vancomycin effect in plasma and tissues against MRSA. **Results**: A PBPK model was successfully built for both healthy volunteers and septic patients. The tissue concentrations were found to be significantly lower than plasma concentrations. The studied strain of MRSA was found to have an MIC of 2 µg/mL, and the PD model described the EC50 as 1.05 µg/mL. The PBPK and PD models were successfully combined, and septic patients infected with MRSA strains with MIC of 2 µg/mL had effective treatment response. However, septic patients infected with MRSA strains with MICs of 4 µg/mL and 8 µg/mL did not have adequate response to vancomycin treatment. **Conclusions**: In septic patients, response was limited against resistant MRSA strains. These findings should be considered hypothesis-generating and interpreted with caution, underscoring the need for individualized approaches and rigorous monitoring.

## 1. Introduction

Infectious diseases are caused by various microorganisms, such as bacteria, viruses, protozoa, fungi, or parasites. These clinical conditions can be acquired by the body in multiple ways, including direct contact with an infected individual, ingestion of contaminated food and/or water, insect bites, wounds, or contact with blood [1]. In 2020, infectious diseases accounted for 17% of deaths in Brazil, making them the second leading cause of mortality in the country and a significant public health issue. The prevalence of infectious diseases is linked to a lack of basic sanitation, precarious health services, poverty, low educational attainment, and other related factors [2].

One of the main complications of infectious diseases is septicemia, characterized by the presence of bacteria in the bloodstream, triggering a series of homeostatic disturbances [3]. In such cases, antimicrobial administration is recommended within one hour of diagnosis. The determination of the antimicrobial agent and the initial loading dose is performed empirically, based on the patient’s symptoms and the infection’s primary site [4].

Vancomycin (VAN) is one of the treatment options considered, as outlined in the Clinical Guide to Antimicrobial Therapy in Sepsis. It features a loading dose between 25 and 30 mg/kg and a maintenance dose of 15 to 20 mg/kg every 8 or 12 h, with adjustments made according to serum drug levels [5]. However, this dosing regimen’s determination was based on a static pharmacodynamic (PD) parameter, the minimum inhibitory concentration (MIC), as well as the pharmacokinetic (PK) parameter free plasma area under the curve (AUC0-inf), which does not necessarily reflect free concentrations at the site of action [6,7]. Furthermore, septic patients frequently exhibit physiological changes that can alter the PK of drugs [8].

Due to these limitations, physiologically based pharmacokinetic (PBPK) modeling combined with pharmacodynamics is being studied to integrate the physiological characteristics of an organism with the physicochemical, PK, and PD properties of a drug. This approach allows the prediction of free concentrations at biophase, and its integration to the effect evaluated in vitro/in vivo, with the cellular death of organisms over time per administered dose [9].

Abraham (2019) [10] was the first to measure free interstitial concentrations of VAN in septic patients using microdialysis (MD, gold-standard technique to measure unbound concentrations in tissues), revealing incomplete and highly variable tissue distribution. Despite the relevance of these findings, such interstitial data have not yet been incorporated into physiologically based pharmacokinetic (PBPK) models. The only VAN PBPK model developed specifically for septic patients, by Radke et al. (2017) [11], incorporated total plasma concentrations only, and has never been used to predict tissue exposure in this population.

Given the previously discussed limitations, this work proposes the evaluation of free VAN concentrations in the tissues of septic patients after the administration of empirical doses used in hospital practice, as well as the probability of these doses reaching therapeutic targets through a PBPK/PD model. Additionally, it aims to analyze the need for adjustments in VAN dosing for these patients. This represents the first PBPK/PD model to propose the prediction of tissue-free VAN concentrations in septic patients, contributing to more effective and safer therapy.

## 2. Materials and Methods

### 2.1. PBPK Model Building

#### 2.1.1. Software Platforms

PBPK models were developed using a small molecule module in PK-Sim (Open Systems Pharmacology Suite 11.0). Published data used for model validation were extracted using WebPlotDigitalizer [12]. Time-kill curves were modeled using NONMEM (version 7.4, ICON Development Solutions, Ellicott City, MD, USA) and PsN version 4.9.0 software (Perl-speaks-NONMEM, Uppsala, Sweden). Pharmacokinetic parameters were derived through non-compartmental analysis (NCA) using PK-Sim or PKanalix (version 2024R1, 8 SimulationsPlus Inc., Lancaster, CA, USA). Tidyverse library for R program (version 4.2, The R Foundation for Statistical Computing, Vienna, Austria) was used to process data.

#### 2.1.2. Healthy Volunteers

Literature data from five previous studies covering VAN plasma concentration in healthy patients were used to validate the model: Blouin et al., Boeckh et al., Cutler et al., Healy et al., and Krogstad et al. [13,14,15,16,17]. VAN was downloaded from the PK-Sim compound database and all physicochemical characteristics of the molecule and PK data are summarized in Table 1. Default physiological properties of the human body were used to describe healthy volunteers. Interstitial tissue exposure could not be developed as there are no published MD studies for VAN in healthy volunteers. Partition-coefficients were estimated using the Schmitt method [18] and cellular permeability using Charge dependent Schmitt method. The estimated elimination was performed setting glomerular filtration rate fraction (GFR) as 1. Model evaluation was conducted using average fold error (AFE) (Equation (1)) for observed versus predicted concentrations, with AFE providing insight into the overall bias of these predictions. Additionally, the fold error (FE) (Equation (2)) was used for observed versus predicted clearance (CL), volume of distribution (Vd or Vss), maximum concentration (C_max_) and area under curve (AUC). The acceptance criteria were within 0.5 to 2.0-fold for both metrics.

(1) $$AFE=10^{\frac{1}{n}\sum {log}_{10}\left(\frac{C_{pred}}{C_{obs}}\right)}$$

(2) $$FE=\frac{{PKparameter}_{obs}}{{PKparameter}_{pred}}$$

#### 2.1.3. Septic Patients

The literature-based-sepsis population model developed by Radke et al [11]. was employed to predict the physiological changes in septic patients and their impact on drug disposition within the PBPK framework. It includes changes in blood flow, body composition, protein binding, cardiac output, and creatinine clearance. This model was used as the initial framework, since protein binding and clearance were modified based on data from De Cock et al. [19] and Matzke et al. [20], respectively. Additionally, the study by Abraham et al. [10] evaluated concentrations of VAN in plasma and subcutaneous free concentrations by MD in septic patients, which was employed to validate the model. As creatinine clearance for patients was reported in that study, this value was used to calculate the renal clearance of septic patients with the Matzke et al. [20] model. Endothelial permeability (Pend) of subcutaneous tissue was optimized using the Levenberg-Marquardt algorithm [21] to accurately predict unbound interstitial concentrations. Model evaluation and acceptance criteria were the same used in the healthy volunteer model.

### 2.2. Pharmacodynamic Model Building

#### Methicillin-Resistant *Staphylococcus aureus* (MRSA)

VAN bactericidal effect was evaluated in a strain of MRSA (ATCC 43300) through time-kill curves. Minimum inhibitory concentration (MIC) was determined by broth microdilution method. After incubation at 35 °C for 24 h, visual reading was performed, with the MIC being defined as the lowest concentration capable of visibly inhibiting bacterial growth. Sterile flasks containing 20 mL of Mueller-Hinton broth were inoculated with 100 uL of bacterial suspension and incubated for 3 h and 30 min at 35 °C to reach the exponential growth phase (log phase). The concentrations used during time-kill experiments represent 0.25, 0.5, 1, 2, 4, 6, and 8 times the MIC for the antimicrobial, alongside a growth control for comparison. Samples were collected at time points 0, 1, 2, 4, 6, 8, 10, 12, and 24 h, diluted in plates, incubated and counted as CFU/mL as described in Dalla Lana et al. [22] and Menezes et al. [23]. Killing curve experiments were performed in triplicate for each concentration. Satisfactory bactericidal activity was defined as a reduction of ≥2 log10 CFU/mL compared to the untreated growth control. Based on the data obtained from the time-kill study, a PD model was developed to describe the relationship between observed effect and drug concentration, incorporating the Emax effect model equation and the adaptive resistance (AR) model previously described by Vera-Yunca et al. [24]. Briefly, the model describes bacterial growth using active (A) and dormant (D) bacteria compartments with a transfer rate from A to D (kad) and from D to A (kda), a growth rate in A state, and a natural death rate in both states. The drug effect acts in A state and is modulated by AR. This modulation is represented by two compartments, ARon and ARoff, linked by a kon rate, which is activated by drug concentration in the system. Exposure to the drug triggers a shift of bacterial populations from the ARoff to the ARon state. As the ARon compartment accumulates, it progressively modulates resistance by elevating the half maximal effective concentration (EC50) linearly, which reduces drug potency, requiring greater concentrations to achieve 50% of the maximal bactericidal activity. All model equations are discussed in the Supplementary File Section S1.

(3) $$Effect=\frac{E_{max}\times C^{\gamma }}{{EC}_{50}^{\gamma }+C^{\gamma }}$$

where effect is the measured PD response, E_max_ is the maximum effect, C is the drug concentration, EC50 is the drug concentration that produces 50% of the maximum effect, and γ is the Hill coefficient for sigmoidal curve.

Model robustness was assessed using the sampling importance resampling (SIR) method (n = 1000), by comparing the original parameter estimates with the median values and 95% confidence intervals obtained from the replicates.

### 2.3. PBPK/PD Model Building

**Figure 1:**
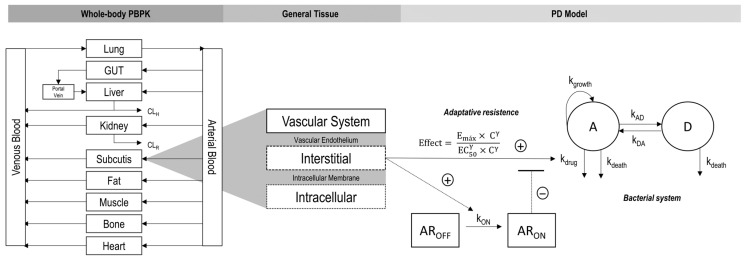
Schematic workflow integrating the physiologically based pharmacokinetic (PBPK) model with the pharmacodynamic (PD) model to predict the effect of vancomycin (VAN) against methicillin-resistant *Staphylococcus aureus* (MRSA). The whole-body PBPK model (**left**) represents drug distribution across major organs and tissues; GUT is gastrointestinal tract; CL_H_ is hepatic clearance; CL_R_ is renal clearance. In the general tissue structure (**center**), drug movement occurs from the vascular system across the vascular endothelium into the interstitial space and through the intracellular membrane into the intracellular space. The interstitial compartment provides unbound drug concentrations for input into the PD model. The PD model (**right**) incorporates adaptive resistance (AR) of MRSA in the presence of VAN, where the antimicrobial effect is described by the Emax model. The bacterial system is divided into active (A) and dormant (D) populations; Emax is the maximum bactericidal effect, EC50 is the drug concentration producing 50% of Emax, C is the unbound drug concentration in interstitial compartment, γ is the Hill coefficient, k_growth_ is active bacterial growth rate, k_death_ is natural bacterial death rate, k_drug_ is bacterial kill rate driven by VAN concentration, k_AD_ and k_DA_ are the transfer rates between active and dormant states, AR_OFF_ and AR_ON_ represent the inactive and active AR states, respectively, with activation driven by VAN concentration at rate k_ON_.

The final PBPK model was coupled to the in vitro PD model describing VAN activity against MRSA, allowing quantitative prediction of bacterial kill-time profiles in multiple tissues. The overall modeling workflow and integration steps are detailed in Figure 1.

Simulations were carried out for the kidney, lung, liver, and subcutaneous tissue, considering their clinical relevance as common infection sites in sepsis. Organ-specific physiological alterations induced by sepsis were incorporated into the model to reflect pathophysiological changes that impact drug distribution, particularly affecting capillary permeability and blood supply. A virtual population of 100 adult subjects with sepsis was generated in PK-Sim. The population had a 1:1 male-to-female ratio, an age range of 50–70 years, and body weights assigned according to the default PK-Sim adult distributions for this age range. Renal clearance was determined from the GFR calculated internally by PK-Sim based on age- and weight-dependent physiological scaling, which accounts for maturational changes, aging effects, kidney size, and body weight. This approach resulted in natural interindividual variability in GFR within the simulated cohort. The demographic characteristics of the generated population are represented in Figure S1. Additionally, the optimized Pend, previously calibrated based on available MD data, was applied to the perfusion-limited tissues under evaluation. The dosing regimen followed the ILAS protocol [5] (Table 2) for general and maximum dosing in sepsis. Also, we performed simulations with continuous infusion to see if this regimen could improve PD response. The resulting interstitial concentration–time profiles were integrated into the PD model to estimate VAN efficacy at the site of infection, providing a mechanistic assessment of the exposure–response relationship under septic conditions.

## 3. Results

### 3.1. Healthy Volunteers PBPK Model

A PBPK model was successfully developed to characterize VAN plasma concentration data. Five studies were found to validate the PBPK model for healthy patients, as detailed in Table S1, however, two of them were only partially included due to internal protocol modifications. Boeckh et al. featured VAN pharmacokinetics in five males and five females aged 20 to 50 years that received a single dose of 500 mg or 1000 mg in one hour [14]. However, due to adverse effects, the administration protocol for the 1000 mg dose was modified during the experiment from a one-hour to a two-hour infusion. Consequently, only the 500 mg dose data were used to validate our model. Cutler et al. studied VAN pharmacokinetics in six young males and six elderly males after a one-hour intravenous infusion of 6 mg/kg [15]. Nonetheless, as one 13-year-old patient received a 30 min infusion instead, the younger patients were excluded from the validation of our model, and an elderly population was used to better simulate the events. All average fold error values remained within the acceptance criteria (Table 3). The goodness-of-fit plot can be seen in Figure S2. Additionally, the model successfully predicted the pharmacokinetic parameters, as all fold error values remained between 0.5 and 2.0 (Table S2).

### 3.2. Septic Patients PBPK Model

**Figure 2:**
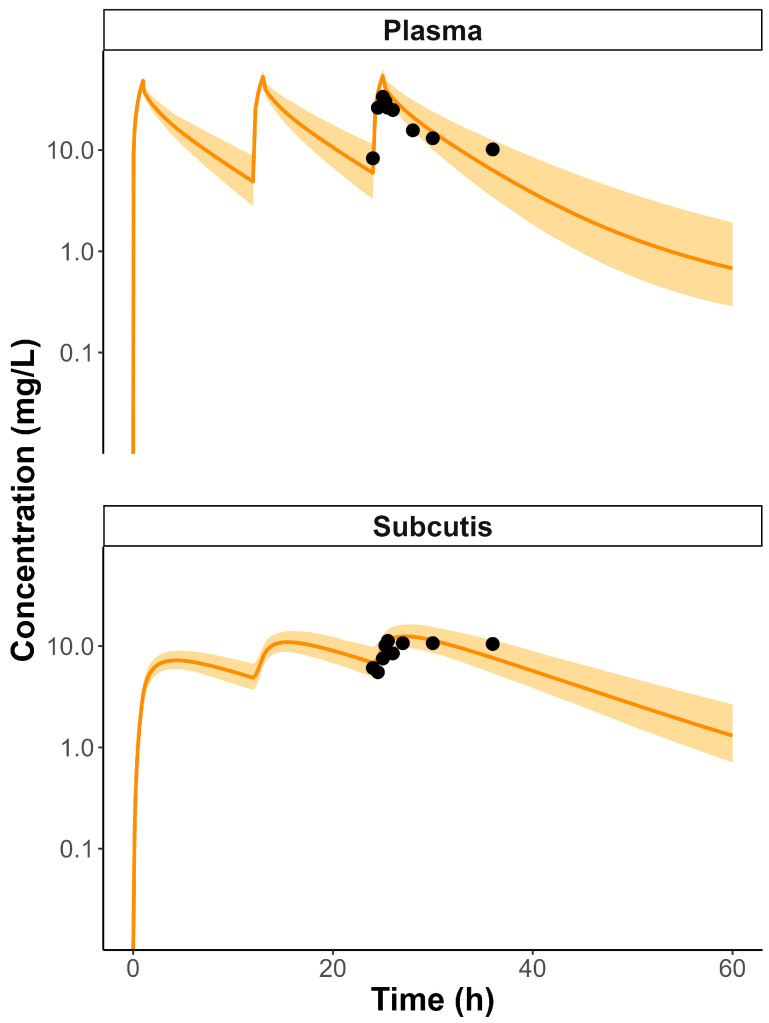
Observed and model-predicted arterial plasma and subcutaneous concentration–time profiles for septic patients. Black dots represent observations from the Abraham et al [20] study. The orange solid line shows the model prediction, and the orange-shaded area represents the 95% confidence interval of the prediction.

One study was found to validate the model, containing arterial plasma and subcutaneous MD data, as detailed in Table S2 [20]. The samples were collected following the third VAN dose administration, a scenario which was subsequently simulated within the model. Evaluation of the PBPK models in plasma and interstitial fluid (ISF) results indicate that it successfully captures the pharmacokinetics of VAN in septic patients, although the Vd was found to be underpredicted in the plasma model (Table S3). The predicted concentration–time profiles are shown in Figure 2 and compared with the observed data from Abraham et al. [20]. The goodness-of-fit plot can be seen in Figure S3. The AFE for the models were found to meet the acceptance criteria, with plasma and ISF AFE values of 1.1655 and 1.1455, respectively.

### 3.3. PD Model

**Figure 3:**
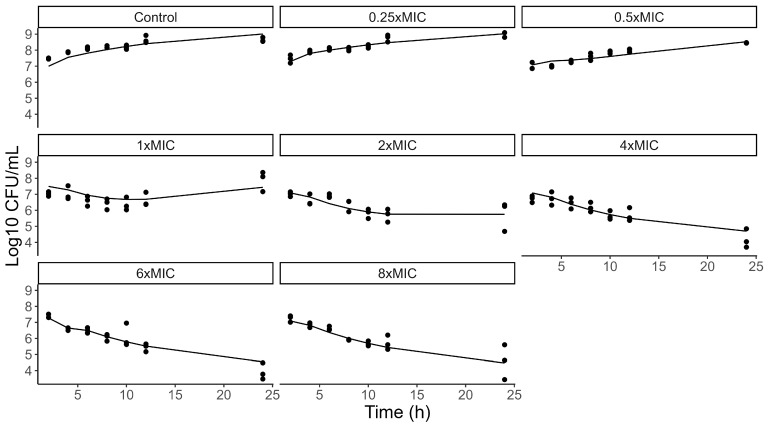
Log10 CFU/mL predicted by the PD model (continuous line) versus observed from the time-kill study raw data (black dots).

The MIC of VAN for the MRSA strain was determined to be 2 µg/mL, classifying it as susceptible. The resulting time-kill data are presented in Figure 3. A model for this data was successfully developed, exhibiting SIR confidence intervals and median values consistent with the model’s effect parameters (Table 4). The bacteria exposed to the 1xMIC concentration exhibited regrowth after 12 h. This outlined the necessity of describing the AR model as presented in the Section 2. With this model we described an EC50 of 1.05 mg/L of VAN in the absence of resistance, for the 2 µg/mL MIC. To enable the creation of a PD model for other MICs, the Schmidt et al. model [25] was utilized to describe the interrelationship of pharmacodynamic parameters, as described in Equation (4). The calculated EC50 values were 2.1 and 4.2 mg/L for 4 and 8 µg/mL MICs, respectively. The visual predictive check of the model is presented in the Supplementary Material (Figure S4).

(4) $$MIC={\left(\frac{d}{E_{max}-d}\right)}^{\frac{1}{\gamma }}\times EC50$$

where MIC is the minimum inhibitory concentration of the bacteria, d is a constant that combines the bacterial growth rate constant (*k*0), the initial inoculum (*N*0), the bacterial count at the MIC turbidity limit (NMIC), and the fixed time point (*t*) of MIC determination, EC50 is the drug concentration that produces 50% of the maximum effect, Emax is the maximum effect, and γ is the Hill coefficient for sigmoidal curve.

### 3.4. PBPK/PD Model

**Figure 4:**
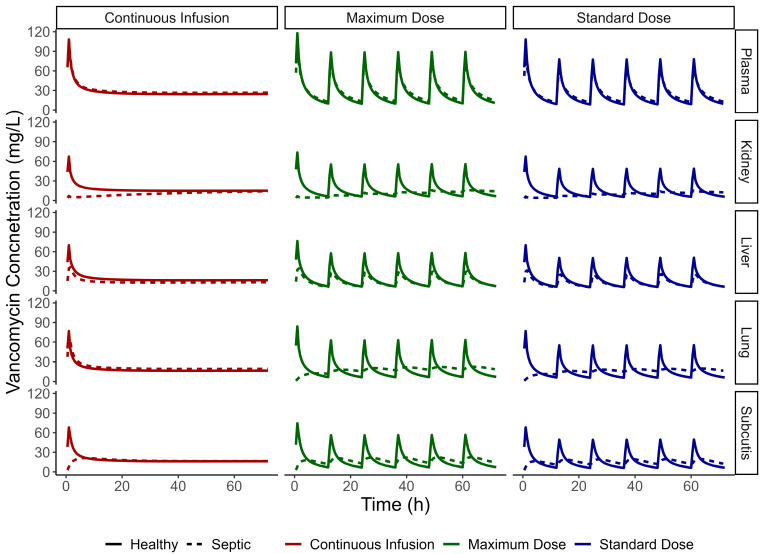
Comparison of VAN concentrations versus time in plasma, kidney, liver, lung, and subcutis in healthy volunteers (solid line) and septic patients (dashed lines) for the intermittent infusion regimens of 17.5 mg/kg (blue), 20 mg/kg (green), and the continuous infusion protocol of 35 mg/kg/24 h (red).

**Figure 5:**
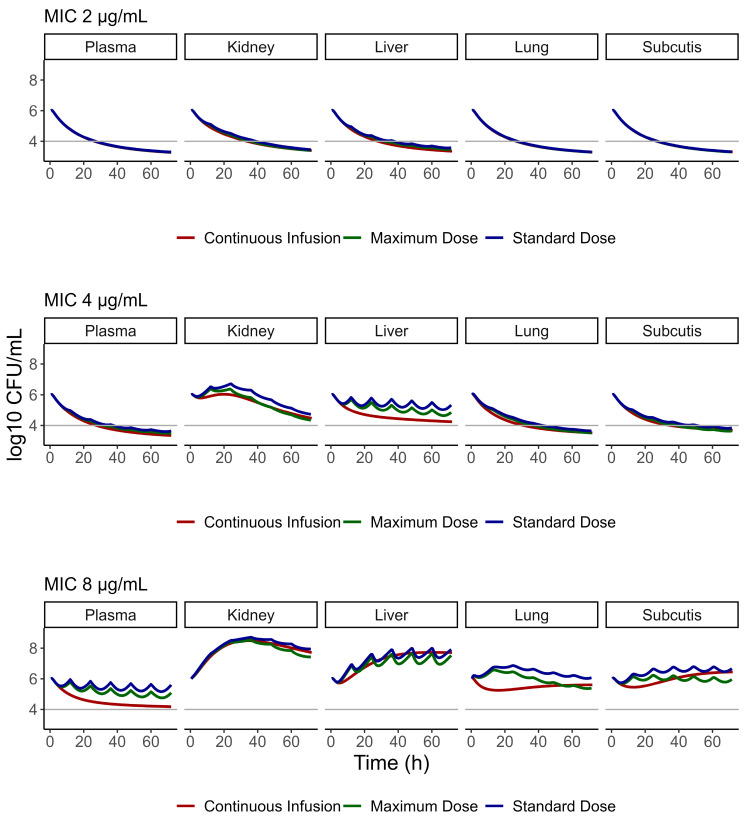
Bacterial cell death versus time for septic patients in plasma, kidney, liver, lung, and subcutis for MRSA strains with MICs of 2, 4, and 8 µg/mL in the intermittent infusion protocols of 17.5 mg/kg and 20 mg/kg q12h (blue and green, respectively), and in the continuous infusion protocol of 35 mg/kg q24h (red). The gray line represents the 2-log reduction of the bacterial load.

Permeability values for perfusion-limited tissues (kidney, liver, and lung) were described using PK-Sim parameters for subcutaneous tissue. The estimated concentrations in all tissues can be seen in Figure 4. For MRSA infections with an MIC of 2 µg/mL, our results indicate that VAN doses of 17.5 mg/kg, 20 mg/kg, and 35 mg/kg/24h were effective in septic patients, achieving bacterial cell death across all simulated tissues within the first 24 h (Figure 5). In contrast, septic patients that received the simulated doses of VAN and were facing MRSA strains with an MIC of 4 µg/mL had an adequate response in plasma, lung, and subcutis within the initial 24 h of treatment. However, the kidney tissue showed an insufficient response during that same period, marked by a significant increase in bacterial growth. Furthermore, the liver tissue’s response proved to be inefficient, as it did not produce adequate bacterial cell death. A continuous infusion protocol resulted in a better response than intermittent doses in these tissues. However, the response was not pronounced enough to achieve adequate cell death. Additionally, septic patients infected with MRSA strains with an MIC of 8 µg/mL treated with either VAN intermittent administration protocol or continuous infusion did not produce adequate response in any simulated tissue or in plasma.

These results confirm that plasma levels are not an adequate surrogate to predict the free tissue levels reached in septic patients. Thus, PK/PD index based on plasma concentrations may not be adequate to ensure successful therapy in generalized infections, or even be used to describe effectiveness of bacterial death in site of action.

## 4. Discussion

This study evaluated the response to conventional VAN therapy for MRSA (MICs of 2, 4, and 8 µg/mL) in the tissues most impacted by sepsis in patients. The model was first validated using healthy volunteers’ data then sepsis-related physiological changes in system parameters, protein binding, and renal clearance as proposed by Radke et al. [11], De Cock et al. [19], and Matzke et al. [20], respectively, were applied to simulate VAN PK. The septic population model was validated using subcutaneous ISF data from microdialysis studies. This application allowed assessment of the model’s generalizability and supported the relevance of the physiological scaling factors proposed by Radke et al. [11], together with the additional parameter adjustments from previous studies [19,20], in predicting drug distribution under septic conditions.

Even though the model showed good overall performance, especially in predicting ISF concentrations, we observed an underprediction (FE 2.04) of the Vd in patients with sepsis. This may reflect the complex and heterogeneous pathophysiological changes associated with sepsis, such as increased capillary permeability, fluid shifts, and alterations in tissue binding. A previous study using the same patient population also reported an underprediction of Vd, reinforcing the idea that additional refinement is needed to better describe drug distribution in the context of sepsis [26]. Nevertheless, the AFE (1.17) for plasma remained within the acceptance criteria, suggesting that, despite this limitation, the model predictions are still reliable for its use in simulations.

Our analysis found adequate response in all VAN regimens in patients infected with MRSA with an MIC of 2 µg/mL. However, patients that were facing MRSA with an MIC of 4 µg/mL or 8 µg/mL did not produce an adequate response in any VAN regimen. The estimated AUC and AUC0-24 values for each regimen can be found in Tables S4 and S5, respectively. Our findings contradict the consensus guidelines that recommend a VAN reference daily AUC between 400 and 600 mg.h/L. Such recommendations, however, are predicated on the assumption of an MRSA broth with an MIC of 1 µg/mL [27,28]. Consequently, it would be inappropriate to refer exclusively to these guidelines in our context, where higher MICs (4–8 µg/mL) do not match the underlying assumptions. This highlights a significant clinical challenge in managing MRSA infections with elevated MICs, suggesting that current standard VAN dosing strategies may be insufficient for achieving adequate antimicrobial exposure in these critical cases. Tissue concentrations were significantly lower than plasma concentrations which led to insufficient bacterial cell death in the kidney and liver of the patients infected with a 4 µg/mL MIC MRSA. This reduced the oscillation in VAN concentrations in these patients, which can also explain why continuous infusion performed better in most simulated conditions. This also indicates that plasma AUC should not be the sole reference for therapeutic monitoring in sepsis.

Despite the fact that sepsis is often linked with bacteremia, both events do not always occur simultaneously, and blood-culture negative sepsis is common [29]. This can further corroborate the explanation of why antimicrobial plasma concentrations should not be the gold standard for therapeutic monitoring in sepsis.

Tissue AUC for liver and kidney was found to be lower in the septic population compared to the healthy volunteer population, despite the significantly reduced elimination (Table S4). This can be explained by the reduced permeability to these tissues, as perfusion is set as augmented in patients with sepsis in PK-Sim, with the exception of subcutaneous tissue. However, the permeability of perfusion-limited tissues in the healthy volunteer population was set as 100 cm/min, which is a PK-Sim standard value for perfusion-limited tissues, as there were not any studies regarding VAN microdialysis in healthy volunteer tissues. Thus, the concentrations in healthy volunteer tissues may be overpredicted in this study, as PK-Sim standard permeability is very high.

For patients with sepsis, an optimal PK/PD index of AUC/MIC for VAN is suggested in the range of 400 to 600. To ensure safety and minimize toxicity risks, it is recommended that the VAN plasma AUC should be maintained below 800 mg.h/L, and trough concentrations should not exceed 15 mg/L [27]. However, simulations showed that even when the dosing regimens surpassed the recommended plasma AUC0-24 of 600 mg.h/L (Table S5), the therapeutic response was inadequate against resistant MRSA strains. Therefore, despite reaching or exceeding recommended therapeutic levels, VAN may not be effective against resistant MRSA strains. Current data does not support therapeutic monitoring of peak concentrations. The inadequacy of therapeutic response against resistant MRSA strains, even with VAN levels considered optimal, is consistent with the literature, which indicates that achieving a targeted AUC/MIC may not be feasible with conventional dosing regimens if the bacterial MIC is 2 µg/mL or greater [27]. The continuous infusion regimen’s AUC0-24 has exceeded the recommended maximum of 800 mg·h/L for minimizing toxicity risks. This suggests that patients who received this regimen may be at higher risk for nephrotoxicity (and potentially ototoxicity) than those who received the intermittent infusion regimens.

Our model estimated an Emax of 0.061 h^−1^ for MRSA, even when exposed to VAN concentrations eight times above the MIC. This relatively slow bactericidal activity resulted in a half-kill time of 11 h. This prolonged death time suggests that MRSA might be developing adaptive resistance, even at what should be very effective drug concentrations. We were able to estimate the AR behavior and capture a progressive reduction in antimicrobial effect with increasing concentrations, indicating a concentration-dependent AR pattern [30].

We selected MRSA because it is a clinically relevant pathogen in sepsis, frequently associated with unfavorable outcomes, and VAN remains one of the primary therapeutic options for its treatment [5]. The aim was to use a representative and challenging target to demonstrate the integration of a validated PBPK model with an in vitro PD model, rather than to encompass all microorganisms potentially involved in sepsis. The PBPK/PD framework described here can be adapted to other pathogens by incorporating their specific pharmacodynamic parameters. In polymicrobial sepsis scenarios, this approach could be extended to simulate multiple pathogens simultaneously or evaluate antimicrobial combinations, supporting the development of more individualized therapeutic strategies.

Although VAN is a highly hydrophilic compound, a LogP of 2.23 was a better fit to build the PBPK model. This value of lipophilicity was employed in other PBPK models. A possible explanation for this is the back-to-back dimerization of this compound in solution, where polar groups become hidden, thus lowering the molecule’s apparent hydrophilicity [31,32].

The time-kill curve analysis was conducted with a single MRSA strain, which represents a possible limitation regarding the comprehensive understanding of VAN’s activity against the broader spectrum of MRSA isolates. This is particularly relevant given our hypothesis that the traditional plasma-derived PK/PD index may not adequately reflect VAN tissue concentrations in sepsis, and strain-specific differences in tissue penetration or response could exist. However, our study’s primary objective was specifically defined to test an initial hypothesis, rather than to provide a comprehensive analysis across all possible strain variations.

Another possible limitation of our study is that it disregards the immune system’s effect in combating the MRSA. At first, this might suggest we are underestimating VAN’s effect. However, since our PD parameters were generated from in vitro experiments using a low bacterial inoculum, adding neutrophil activity to the model without adjusting for the higher bacterial loads seen in actual infections could end up overstating the drug’s efficacy. Therefore, despite the absence of immune components, the model still provides a reliable representation of the drug’s PD behavior in this controlled setting.

Additionally, the scope of the source data is restricted, relying on a limited number of MD studies for tissue PK, which may not fully capture the variability observed in different clinical settings. Furthermore, no external validation was performed with independent datasets, meaning that the accuracy of the predictions outside the modeled scenarios remains unverified. Consequently, extrapolation of these findings to other clinical contexts should be approached with caution until supported by broader and more diverse datasets.

## 5. Conclusions

In conclusion, our findings demonstrate that VAN concentrations in critically infected tissues were lower than plasma concentrations, highlighting that plasma levels may not be adequate for measuring drug exposure at the site of infection for therapeutic drug monitoring in septic patients. Furthermore, under empiric dosing regimens for sepsis complicated by MRSA infections, adequate therapeutic response, as determined by bacterial cell death, was observed only when the infecting strain was classified as susceptible (MIC ≤ 2 µg/mL). Treatment efficacy was limited against less susceptible or resistant MRSA strains.

These results should be regarded as hypothesis-generating and interpreted with caution, given the reliance on limited datasets. Importantly, the findings specifically reflect the modeled interaction between VAN and bacteria, and therefore require confirmation in broader, more diverse clinical datasets. Nevertheless, this work represents an important first step toward predicting free interstitial concentrations of VAN at the site of infection. By refining our ability to simulate drug behavior in infected tissues, this approach has the potential to support the development of more individualized and physiologically informed dosing protocols of VAN in the future by simulating new scenarios.

## Figures and Tables

**Table 1:** Input parameters for the VAN healthy volunteers, septic patients’ plasma and subcutaneous PBPK model.

	Healthy Volunteers	Septic Patients	
Parameter	Value	Value	Reference
Molecular weight (g/mol)	1449.30	1449.30	PK-Sim database
Log P	2.23	2.23	PK-Sim database
pKa	2.18 (acid), 7.75 (base), 8.89 (base)	2.18 (acid), 7.75 (base), 8.89 (base)	PK-Sim database
Solubility at pH 7 (mg/L)	225	225	PK-Sim database
Fraction unbound	0.67	0.72	[19]
Binding protein	Albumin	Albumin	PK-Sim database
Has halogens	No	No	PK-Sim database
Partition coefficients	Schmitt	Schmitt	PK-Sim [18]
Cellular permeabilities	Charge dependent Schmitt	Charge dependent Schmitt	PK-Sim [18]
P plasma ↔ interstitial (cm/s)	100 ^#^	6.00 × 10^−7^	Optimized by PK-Sim
Renal CL (mL/min/kg)	0.91 *	0.80	[20]
Hepatic CL (mL/min/kg)	0.07	0.07	[11]

^#^ Assuming instantaneous distribution by PK-Sim. * This value was estimated by PK-Sim given that the GFR fraction was set to 1.

**Table 2:** Dosing regimens simulated in the final PBPK/PD model.

Protocol	Loading Dose (mg/kg)	Maintenance Dose (mg/kg)	Time of Infusion (h) [Frequency]	Treatment Duration (Days)
Continuous infusion	27.5	35	24 [continuous]	10
Typical sepsis dose	27.5	17.5	1 [q12h]	10
Highest sepsis dose	30	20	1 [q12h]	10

**Table 3:** Average fold error of the model across validation studies.

Study	Dose	AFE Value
Blouin et al. [13]	1000 mg	0.9798
Boeckh et al. [14]	500 mg	1.1468
Cutler et al. [15]	6 mg/kg	1.2989
Healy et al. [16]	500 mg	1.0282
Healy et al. [16]	1000 mg	1.0322
Krogstad et al. [17]	500 mg (patient 1)	1.3674
Krogstad et al. [17]	500 mg (patient 2)	1.1475
Krogstad et al. [17]	700 mg	1.2503
Krogstad et al. [17]	1000 mg	1.1581

**Table 4:** PD models final parameters with SIR medians and 95% confidence interval.

Effect Parameter	Unit	Value	RSE (%)	SIR (95% CI)
k_growth_	h^−1^	1.96	1.8	1.95 (1.89–2.01)
Hill factor		5.74	3.4	5.72 (5.41–6.05)
Maximum effect	h^−1^	0.061	0.5	0.062 (0.060–0.065)
EC50 _in absence of AR_	mg/L	1.05	0.7	1.05 (1.03–1.06)
Maximum bacteria	Log CFU/mL	8.94	1.8	8.93 (8.66–9.21)
k_ON_	h^−1^	0.021	3.7	0.022 (0.021–0.023)
k_DA_	h^−1^	0.022	3.7	0.022 (0.020–0.023)
k_death_	h^−1^	1.85	1.4	1.85 (1.80–1.89)
Slope		3.24	1.4	3.23 (3.17–3.31)
Proportional error	%	0.16	13.6	0.16 (0.13–0.19)

## Data Availability

Model files and used data are available by reasonable request.

## Supplementary Materials

The following supporting information can be downloaded at: https://www.mdpi.com/article/10.3390/pharmaceutics17091111/s1, Section S1: PD Model Equations; Table S1: Summary of study designs; Figure S1: Demographic distribution of the virtual septic population; Figure S2: Goodness-of-fit plot for the PBPK healthy volunteers model; Table S2: Fold error parameters across validation studies; Table S3. Summary of the Abraham et al., 2019 [6]; Figure S3: Goodness-of-fit plot for the PBPK septic patients model; Table S4: Fold error of the parameters across validation of Abraham et al., 2019 [6]; Figure S4: Visual predictive check of the MRSA time-kill curve PD model; Table S5: Comparison of C_max_ and AUC parameters.

## References

[1] (2010). Brasil. Ministério da Saúde. Secretaria de Vigilância em Saúde. Doenças infecciosas e parasitárias: Guia de Bolso. 8. ed. Brasília, DF.. https://www.gov.br/saude/pt-br/centrais-de-conteudo/publicacoes/svsa/doencas-diarreicas-agudas/doencas-infecciosas-e-parasitarias_-guia-de-bolso.pdf/view.

[2] da Silva E.L.M., dos Santos S., Torquati A., Araújo C., Brandão F. (2022). Why are infectious and parasitic diseases among the leading causes of death in Brazil?. Res. Soc. Dev..

[3] Santos-Borges K.T., Henz P., de Araújo B.V. (2023). The influence of sepsis on antimicrobials tissue penetration: The use of microdialysis technique to access free drug distribution. Braz. J. Pharm. Sci..

[4] Strich J.R., Heil E.L., Masur H. (2020). Considerations for Empiric Antimicrobial Therapy in Sepsis and Septic Shock in an Era of Antimicrobial Resistance. J. Infect. Dis..

[5] Latin American Sepsis Institute (2022). Practical Guide to Antimicrobial Therapy in Sepsis.

[6] Stanford Health Care (2023). SHC Vancomycin Dosing Guide [S. l.]. https://med.stanford.edu/content/dam/sm/bugsanddrugs/documents/antimicrobial-dosing-protocols/SHC%20Vancomycin%20Dosing%20Guide.pdf.

[7] Meibohm B., Derendorf H. (1997). Basic concepts of pharmacokinetic/pharmacodynamic (PK/PD) modelling. International J. Clin. Pharmacol. Ther..

[8] Girdwood S.T., Tang P., Fenchel M., Dong M., Stoneman E., Jones R., Ostermeier A., Curry C., Forton M., Hail T. (2023). Pharmacokinetic parameters over time during sepsis and the association of target attainment and outcomes in critically ill children and young adults receiving ceftriaxone. Pharmacotherapy.

[9] Kuepfer L., Niederalt C., Wendl T., Schlender J., Willmann S., Lippert J., Block M., Eissing T., Teutonico D. (2016). Applied Concepts in PBPK Modeling: How to Build a PBPK/PD Model. CPT Pharmacomet. Syst. Pharmacol..

[10] Abraham J., Sinnollareddy M.G., Roberts M.S., Williams P., Peake S.L., Lipman J., Roberts J.A. (2019). Plasma and interstitial fluid population pharmacokinetics of vancomycin in critically ill patients with sepsis. Int. J. Antimicrob. Agents.

[11] Radke C., Horn D., Lanckohr C., Ellger B., Meyer M., Eissing T., Hempel G. (2017). Development of a Physiologically Based Pharmacokinetic Modelling Approach to Predict the Pharmacokinetics of Vancomycin in Critically Ill Septic Patients. Clin. Pharmacokinet..

[12] Automeris (2023). WebPlotDigitalizer (Version 4.5). https://apps.automeris.io/wpd/.

[13] A Blouin R., A Bauer L., Miller D.D., E Record K., O Griffen W. (1982). Vancomycin pharmacokinetics in normal and morbidly obese subjects. Antimicrob. Agents Chemother..

[14] Boeckh M., Lode H., Borner K., Höffken G., Wagner J., Koeppe P. (1988). Pharmacokinetics and serum bactericidal activity of vancomycin alone and in combination with ceftazidime in healthy volunteers. Antimicrob. Agents Chemother..

[15] Cutler N.R., Narang P.K., Lesko L.J., Ninos M., Power M. (1984). Vancomycin disposition: The importance of age. Clin. Pharmacol. Ther..

[16] Healy D.P., E Polk R., Garson M.L., Rock D.T., Comstock T.J. (1987). Comparison of steady-state pharmacokinetics of two dosage regimens of vancomycin in normal volunteers. Antimicrob. Agents Chemother..

[17] Krogstad D.J., Moellering R.C., Greenrlatt D.J. (1980). Single-dose kinetics of intravenous vancomycin. J. Clin. Pharmacol..

[18] Schmitt W. (2008). General approach for the calculation of tissue to plasma partition coefficients. Toxicol. Vitr..

[19] De Cock P.A.J.G., Desmet S., De Jaeger A., Biarent D., Dhont E., Herck I., Vens D., Colman S., Stove V., Commeyne S. (2017). Impact of vancomycin protein binding on target attainment in critically ill children: Back to the drawing board?. J. Antimicrob. Chemother..

[20] Matzke G., Kovarik J., Rybak M., Boike S. (1985). Evaluation of the vancomycin-clearance:creatinine-clearance relationship for predicting vancomycin dosage. Clin. Pharm..

[21] Levenberg K. (1944). A method for the solution of certain problems in least squares. Q. Appl. Math..

[22] Lana D.F.D., Kaminski T.F.A., Lavorato S.N., Merkel S., Zanette R.A., da Rosa P.D., Staudt K.J., de Araújo B.V., da Costa B., Quatrin P.M. (2021). In vitro pharmacokinetics/pharmacodynamics modeling and efficacy against systemic candidiasis in *Drosophila melanogaster* of a bisaryloxypropanamine derivative. Med. Mycol..

[23] Menezes B., Alves I., Staudt K., Beltrame B., Michelin L., de Araújo B.V., Tasso L. (2021). PK/PD modeling of daptomycin against MRSA and MRSE and Monte Carlo simulation for bacteremia treatment. Braz. J. Microbiol..

[24] Vera-Yunca D., Matias C., Lundberg C.V., E Friberg L. (2025). Model-based translation of the PKPD-relationship for linezolid and vancomycin on methicillin-resistant *Staphylococcus aureus*: From in vitro time–kill experiments to a mouse pneumonia model. J. Antimicrob. Chemother..

[25] Schmidt S., Schuck E., Kumar V., Burkhardt O., Derendorf H. (2007). Integration of pharmacokinetic/pharmacodynamic modeling and simulation in the development of new anti-infective agents—Minimum inhibitory concentration versus time-kill curves. Expert Opin. Drug Discov..

[26] Bahnasawy A.M., Parrott N.J., Gijsen M., Spriet I., Friberg L.E., Nielsen E.I. (2024). Physiologically-based pharmacokinetic modelling in sepsis: A tool to elucidate how pathophysiology affects meropenem pharmacokinetics. Int. J. Antimicrob Agents..

[27] Rybak M.J., Le J., Lodise T.P., Levine D.P., Bradley J.S., Liu C., Mueller B.A., Pai M.P., Wong-Beringer A., Rotschafer J.C. (2020). Therapeutic monitoring of vancomycin for serious methicillin-resistant Staphylococcus aureus infections: A revised consensus guideline and review by the American Society of Health-System Pharmacists, the Infectious Diseases Society of America, the Pediatric Infectious Diseases Society, and the Society of Infectious Diseases Pharmacists. Am. J. Health Syst. Pharm..

[28] Evans L., Rhodes A., Alhazzani W., Antonelli M., Coopersmith C.M., French C., Machado F.R., Mcintyre L., Ostermann M., Prescott H.C. (2021). Surviving sepsis campaign: International guidelines for management of sepsis and septic shock. Intensive Care Med..

[29] Nejtek T., Müller M., Moravec M., Průcha M., Zazula R. (2023). Bacteremia in Patients with Sepsis in the ICU: Does It Make a Difference?. Microorganisms.

[30] Mi K., Zhou K., Sun L., Hou Y., Ma W., Xu X., Huo M., Liu Z., Huang L. (2022). Application of Semi-Mechanistic Pharmacokinetic and Pharmacodynamic Model in Antimicrobial Resistance. Pharmaceutics.

[31] Schäfer M., Schneider T.R., Sheldrick G.M. (1996). Crystal structure of vancomycin. Structure.

[32] Jia Z., O’MAra M.L., Zuegg J., Cooper M.A., Mark A.E. (2013). Vancomycin: Ligand recognition, dimerization and super-complex formation. FEBS J..

