# Supplementary material for: PBPK/PD Model of Vancomycin in Sepsis: Linking Interstitial Exposure in Perfusion-Limited Tissues to MRSA Infection
Source: Pharmaceutics. 2025 Aug 26;17(9):1111. doi: 10.3390/pharmaceutics17091111 (PMC12473409; doi:10.3390/pharmaceutics17091111)
Supplement: Supplementary file 1 [file pharmaceutics-17-01111-s001.zip › pharmaceutics-3806454-supplementary material.pdf]

Supplementary File

S1. PD Model equations

S1.1. Bacterial Growth

$$\frac{dA}{dt} = k_g \times A - k_{AD} \times A - (k_d + Effect) \times A + k_{DA} \times D \qquad \text{(S1)}$$

$$\frac{dD}{dt} = k_{AD} \times A - k_d \times A - k_{DA} \times D \qquad \text{(S2)}$$

$$k_{AD} = (k_g - k_d) \times \frac{A + D}{N_{max}} \qquad \text{(S3)}$$

Where A is the susceptible population of MRSA, D is the dormant bacterial subpopulation, k_g is the bacterial growth rate, k_{AD} is the transfer rate from A to D state modulated by a maximum bacterial capacity (N_{max}), k_{DA} is the transfer rate from D to A state, k_d is the natural death rate with no drug effect and *Effect* is the drug induced bacterial killing.

S1.2. Adaptative Resistance Model

$$\frac{dAR_{off}}{dt} = -k_{ON} \times Conc \times AR_{off} \qquad \text{(S4)}$$

$$\frac{dAR_{on}}{dt} = k_{ON} \times Conc \times AR_{off} \qquad \text{(S5)}$$

In which, the adaptative resistence is represented by two compartments, AR_{off} and AR_{on}, k_{ON} is the rate between AR_{off} to AR_{on} in the presence of VAN concentration. When bacteria are in contact with VAN, there is a transfer of AR_{off} to $AR_{on.}$ The increasing amount inside AR_{on}, drives the resistance effect (AR_{eff}) on EC50, increasing it.

$$AR_{eff} = 1 + Slope \times AR_{on} \qquad \text{(S6)}$$

$$EC50 = AR_{eff} \times EC50_0 \qquad \text{(S7)}$$

Where AR_{eff} are the AR effect on EC50, Slope is the linear coefficient that controls the AR_{eff} on EC50 and $EC50_0$ is the EC50 in absence of AR.

Table S1. Summary of study designs and patient characteristics of the studies in healthy volunteers included in the validation of the PBPK model.

Variable	Blouin et al. [1]	Boeckh et al.[2]	Cutler et al.[3]	Healy et al.[4]	Krogstad et al.[5]
Number of individuals	4	10	6	11	4
Number of females	0	5	0	4	1
Dose	1000 mg	500 mg	6 mg/kg	500 and 1000 mg	500, 700 or 1000 mg
Age (yr)	27.5 (25-30)	27.4 (20.0-50.0)	68.5 (61.0-77.0)	24.7	36.2 (32.0-40.0)
Weight (kg)	77.5 (65.9-89.1)	65.1 (55.0-78.0)	69.0 (60.1-83.8)	66.5	68.6 (56.8-79.5)
Height (cm)	177.5 (175.3-184.2)	NA	174.2 (165.0-186.0)	NA	NA
Creatinine clearance (mL/min/kg)	1.78	1.47	1.41	NA	1.77 (1.51-2.32)
Dose regimen	Single dose	Single dose	Single dose	Multiple dose (five 500 mg q6h and three 1000 mg q12h)	Single dose
Infusion duration (h)	0.67	1	1	1	0.35 (700 mg dose) or 0.5 (500 and 1000 mg dose)

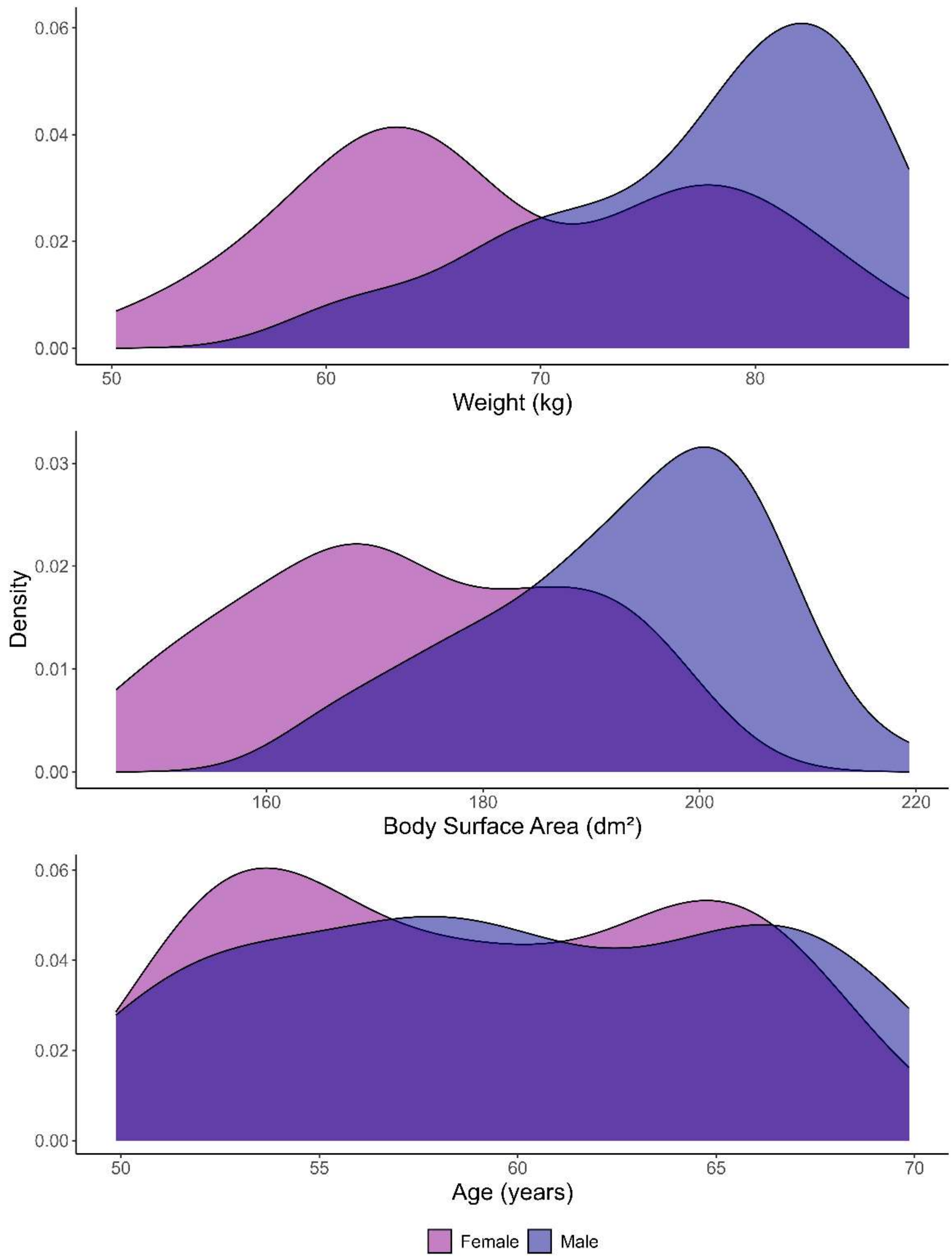

Figure S1. Demographic distribution of the virtual septic population (n = 100) generated in PK-Sim for the simulation scenarios

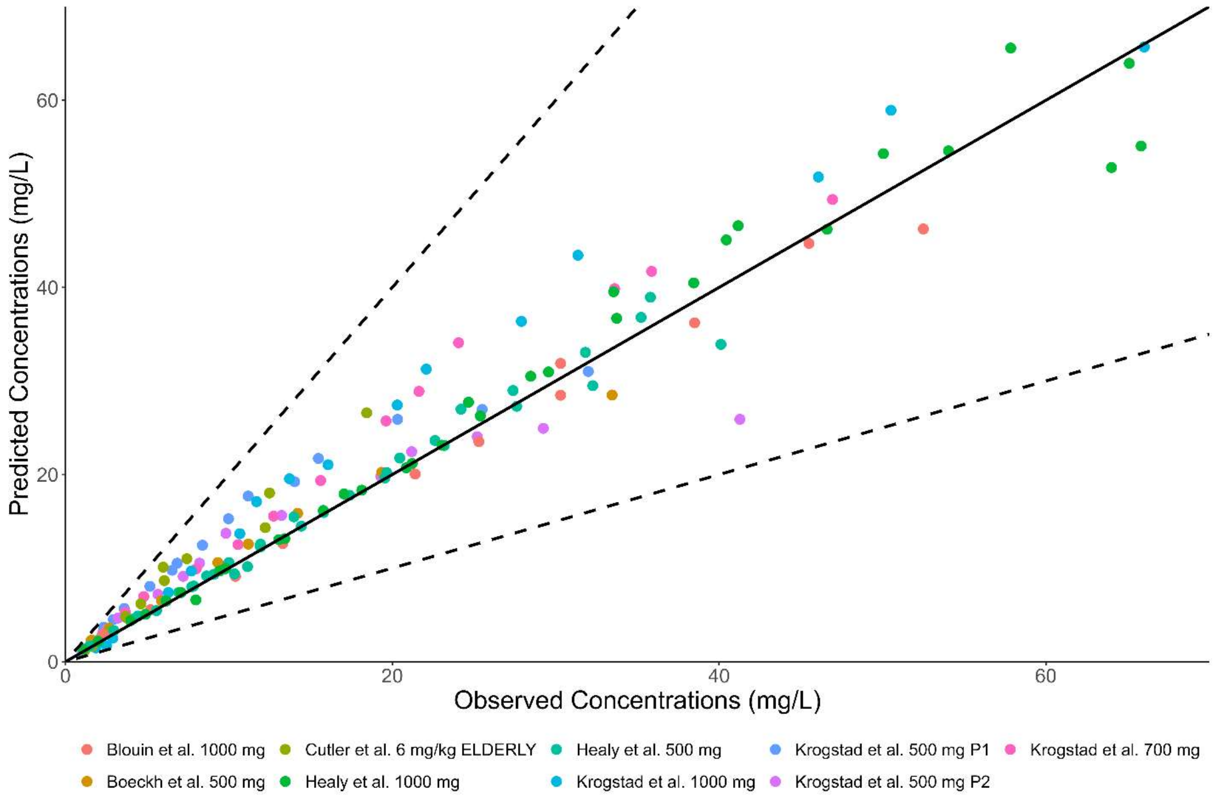

Figure S2. Goodness-of-fit plot for the PBPK healthy volunteers model. The solid line represents the line of identity (perfect prediction), and the dashed lines are the acceptance limits of 0.5- and 2.0-fold deviation.

Table S2. Fold error of the parameters across validation studies. The highlighted* parameters were calculated by non-compartmental analysis in PKanalix.

		**Predicted**				**Observed**				**Fold error**			
**Study**	**Dose**	**CL (L/h/kg)**	**Vd or Vdss * (L/kg)**	**Cmax (mg/L)**	**AUC (mg.h/L)**	**CL (L/h/kg)**	**Vd or Vdss* (L/kg)**	**Cma x (mg/L)**	**AUC (mg.h /L)**	**CL (L/h/ kg)**	**Vd (L/kg)**	**Cmax (mg/L)**	**AUC mg.h/ L)**
Blouin et al.[1]	1000 mg	0.06	0.44*	65.95	247.89	0.065	0.39*	52.50	215.9 4	1.08	0.89		
Boeckh et al.[2]	500 mg	0.064	0.67	29.50	123.64	0.087	0.55	47.0	90.7	1.36	0.82	1.59	0.73
Cutler et al.[3]	6 mg/kg	0.04	0.41*	26.60	138.76	0.052	0.76*	17.7	111.5 8	1.3	1.85	0.66	
Healy et al.[4]	500 mg	0.066	0.31*	38.95	105.46	0.044	0.588*	40.3	116			1.03	1.09
Healy et al.[4]	1000 mg	0.048	0.41*	65.58	236.7	0.045	0.587*	65.7	227			1.00	0.96
Krogstad et al.[5]	500 mg	0.06	0.67	35.74	120.76	0.069	0.77	36.64	1.15	1.15			
Krogstad et al.[5]	700 mg	0.06	0.67	53.76	169.06	0.082	1.25	46.94	1.36	1.86			
Krogstad et al.[5]	1000 mg	0.06	0.67	71.49	241.52	0.065	0.87	66.02	1.05	1.29			

Table S3. Summary of the Abraham et al. study design and included patient population. Although the patients were in a multiple-dose regimen, data collection was limited to a 12-hour period.

**Variable**	**Abraham et al.** [6]
Number of individuals	6
Number of females	2
Dose (mg/kg)	12.25
Age (yr)	55.0 (44.0-67.0)
Weight (kg)	85.0 (81.0-102.0)
Creatinine clearance (mL/min/kg)	90 (83.0-98.0)
Dose regimen	q12h
Infusion duration (h)	1

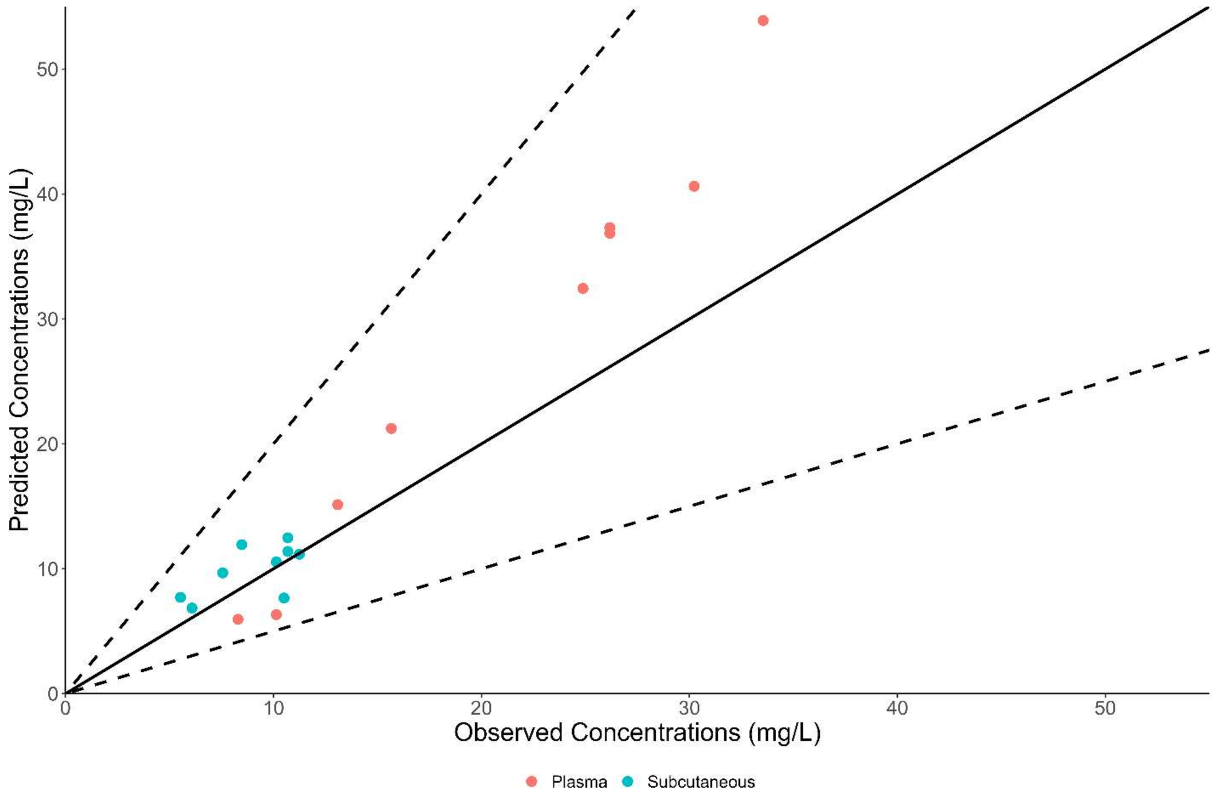

Figure S3. Goodness-of-fit plot for the PBPK septic patients model. The solid line represents the line of identity (perfect prediction), and the dashed lines are the acceptance limits of 0.5 and 2.0.

Table S4. Fold error of the parameters across validation of Abraham et al, 2019. The highlighted parameters were calculated by non-compartmental analysis in PKanalix.

		**Predicted**				**Observed**				**Fold error**			
**Study**	**Tissue**	**CL (L/h/kg)**	**Vd (L/kg)**	**Cmax (mg/L)**	**AUC_{24-36} (mg.h/L)**	**CL (L/h/kg)**	**Vd (L/kg)**	**Cmax (mg/L)**	**AUC_{24-36} (mg.h/L)**	**CL (L/h/kg)**	**Vd (L/kg)**	**Cmax (mg/L)**	**AUC mg.h/L)**
Abraham et al.[6]	Plasma	0.046	0.3	53.90	226.65	0.032	0.61	33.55	190.54	0.69	2.03	0.62	0.84
	ISF	NA	NA	12.51	125.59	NA	NA	11.24	119.6	NA	NA	0.90	0.95

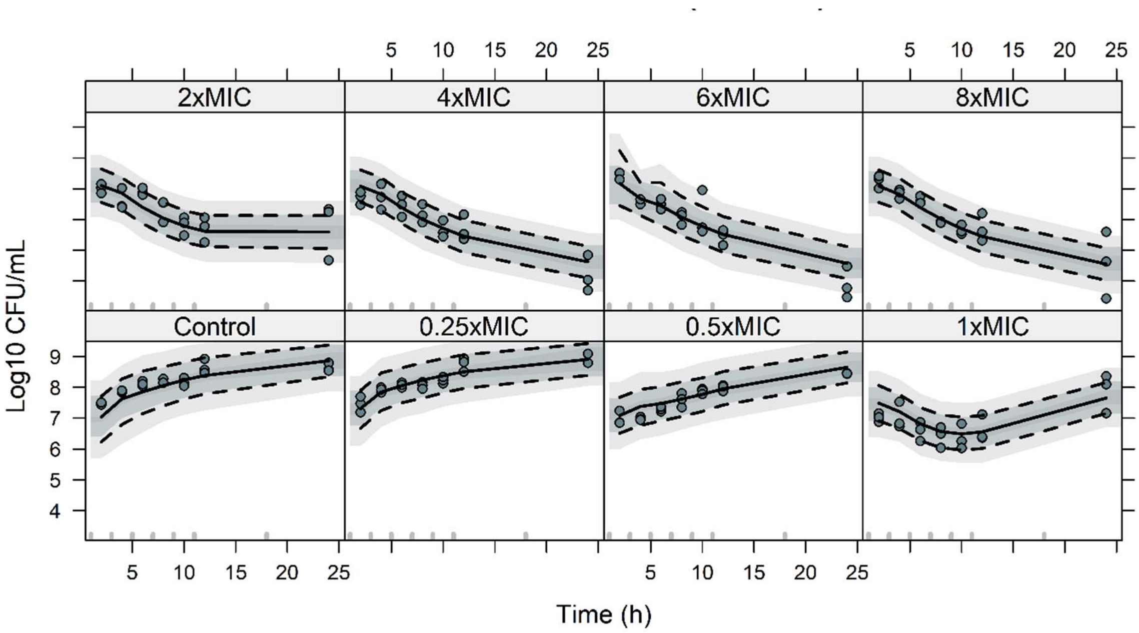

Figure S4. Visual predictive check of the MRSA time-kill curve PD model. VPC is based on 500 simulations and show a comparison of the observations (dots) with the 10th, 50th, and 90th percentiles of simulated profiles (dashed lines and shadow areas).

Table S5. Comparison of C_{max} and AUC parameters for clinically recommended empirical doses in healthy volunteers and septic patients.

		**Healthy**		**Septic**	
**Administration Protocol**	**Tissue**	**Cmax (mg/L)**	**AUC (mg.h/L)**	**Cmax (mg/L)**	**AUC (mg.h/L)**
Continuous infusion	Plasma	108.00	6676.63	109.68	7245.21
	Kidney	67.31	4051.21	17.72	3882.77
	Liver	69.82	4466.05	35.99	3632.12
	Lung	76.96	4487.54	71.49	5234.63
	Subcutis	68.09	4462.60	21.14	4430.80
Typical sepsis dose	Plasma	108.00	6432.94	89.35	6871.00
	Kidney	67.31	3904.54	19.90	3805.82
	Liver	69.82	4314.47	31.34	3467.43
	Lung	76.96	4321.62	20.26	4634.00
	Subcutis	68.09	4301.49	20.44	4282.54
Highest sepsis dose	Plasma	117.82	7326.65	97.48	7825.49
	Kidney	73.43	4447.00	23.06	4400.28
	Liver	76.16	4913.85	34.61	4016.88
	Lung	83.96	4922.01	23.47	5353.04
	Subcutis	74.28	4899.09	23.36	4877.57

References

1. Blouin, R. A. et al. Vancomycin pharmacokinetics in normal and morbidly obese subjects. *AAC*, **1982**, *21*, 575-580. DOI: 10.1128/AAC.21.4.575.
2. Boeckh, M. et al. Pharmacokinetics and serum bactericidal activity of vancomycin alone and in combination with ceftazidime in healthy volunteers. *AAC*, **1988**, *32*, 92-95. DOI: 10.1128/AAC.32.1.92.
3. Cutler, N. R. et al. Vancomycin disposition: the importance of age. *Clin Pharmacol Ther*, **1984**, *36*, 803-810. DOI: 10.1038/clpt.1984.260.
4. Healy, D. P. et al. Comparison of steady-state pharmacokinetics of two dosage regimens of vancomycin in normal volunteers. *AAC*, **1987**, *31*, 393-397. DOI: 10.1128/AAC.31.3.393.
5. Krogstad, D. J. et al. Single-dose kinetics of intravenous vancomycin. *J. Clin. Pharmacol*, **1980**, *20*, 197-201. DOI: 10.1002/j.1552-4604.1980.tb01696.x
6. Abraham, J. et al. Plasma and interstitial fluid population pharmacokinetics of vancomycin in critically ill patients with sepsis. *nt J Antimicrob Agents*, **2019**, *53*, 137-142. DOI: 10.1016/j.ijantimicag.2018.09.021
